# Characterization of the *in Vitro* Metabolic Profile of Evodiamine in Human Liver Microsomes and Hepatocytes by UHPLC-Q Exactive Mass Spectrometer

**DOI:** 10.3389/fphar.2018.00130

**Published:** 2018-02-22

**Authors:** Zhaowei Zhang, Tianzi Fang, Hongyun Zhou, Jie Yuan, Qingwang Liu

**Affiliations:** ^1^Department of Pharmacy, Jinhua Municipal Central Hospital, Jinhua, China; ^2^Anhui Provincial Institute for Food and Drug Control, Hefei, China; ^3^Institute of Technology Innovation, Hefei Institutes of Physical Science, Chinese Academy of Sciences, Hefei, China

**Keywords:** evodiamine, metabolism, human liver microsomes, human hepatocytes, LC-MS

## Abstract

Evodiamine is an indoloquinazoline alkaloid isolated from the fruit of *Evodia rutaecarpa*, which has a wide range of pharmacological effects like anti-tumor and anti-inflammatory effects. This study was intended to investigate the metabolic characteristics of evodiamine in human liver microsomes and hepatocytes by ultra-high performance liquid chromatography coupled with a Q Exactive mass spectrometer. A total of 12 phase I metabolites were detected in human liver microsomes; whereas in human hepatocytes 19 metabolites, including seven phase II metabolites were detected. The structures of the metabolites were characterized based on their accurate masses, fragment ions, and chromatographic retention times. Four metabolites (M1, M2, M5, and M7) were further unambiguously confirmed by matching their retention times, accurate masses, and fragment ions with those of their reference standards. Among these metabolites, 12 metabolites are first identified (M2, M5–M8, M10–M13, and M17–M19). The current study revealed that oxygenation, *N*-demethylation, dehydrogenation, glucuronidation, and GSH conjugation were the major metabolic pathways for evodiamine. This study elucidated the detailed metabolite profiles of evodiamine, which is helpful in predicting *in vivo* metabolism of evodiamine in human and in understanding the elimination mechanism of evodiamine and in turn, the effectiveness and toxicity.

## Introduction

**FIGURE 1:**
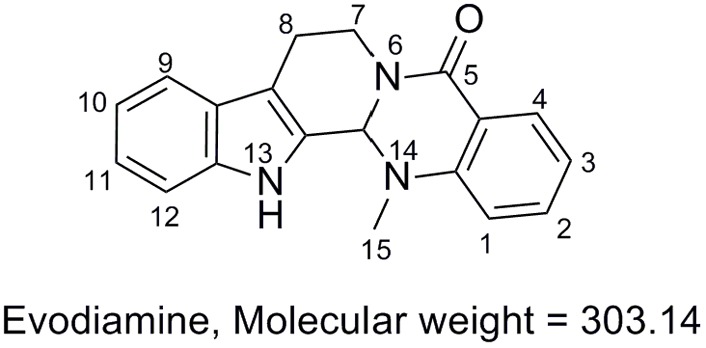
Chemical structure of evodiamine.

*Evodia rutaecarpa* (Juss.) Benth (family of Rutaceae) has been widely used for medicinal purposes in China for treatment of aches and gastrointestinal disorders for thousands of years (Chinese Pharmacopoeia Commission, 2010). Evodiamine (**Figure 1**), an indoloquinazoline alkaloid isolated from the fruit of *E. rutaecarpa*, was found as one of the main active components in this herb medicine. Evodiamine has been reported to function as anti-inflammatory (Chiou et al., 1997; Ko et al., 2007), vasodilatory (Chiou et al., 1996), and anti-obesity agent (Kobayashi et al., 2001). Recently, evodiamine has drawn increasing attention due to its anti-tumor activity by inhibiting proliferation of various cancer cell lines (Liao et al., 2005; Jiang and Hu, 2009), by inducing apoptosis (Zhang et al., 2004), and by inhibiting topoisomerases I and II (Pan et al., 2012).

Previous pharmacokinetic study demonstrated that evodiamine showed very low oral bioavailability (Komatsu et al., 1993), which may be attributed to the extensive metabolism. Komatsu et al. (1993) have reported that evodiamine could be rapidly converted to its metabolites (see also Lee et al., 2017), and its metabolite 10-hydroxyevodiamine showed stronger cytotoxicity than evodiamine (Li et al., 2006). Wen et al. (2014) reported that evodiamine undergo metabolic bioactivation to form its reactive metabolites, and evodiamine is a time-dependent inhibitor of CYP3A4. These results indeed provided some clues in interpreting the mechanism of hepatotoxicity of evodiamine. Although several studies referred to the metabolism of evodiamine have been reported (Sun et al., 2013; Wen et al., 2014), detailed metabolite profiling of evodiamine, especially in human, has not been carried out. Detailed metabolite information is helpful to understand the mechanism of drug action.

In early drug discovery stage, it is hard to get *in vivo* human metabolism data due to the ethics and safety issues. Human liver microsomes and hepatocytes are alternatives. Liver microsomes are the most widely used for drug metabolism studies as they are easy to handle and commercially available. However, there are few limitations (Basu and Shaik, 2016). For example, liver microsomes lack the cell membranes to mimic the physiological environment in hepatocytes. In addition, they have to be supplemented with cofactors such as reduced nicotinamide adenine dinucleotide phosphate (NADPH) or uridine 5′-diphosphate glucuronic acid. By contrast, hepatocytes are more suitable for metabolism study as they overcome the limitations above and they represent a more complete system to study drug phase I and phase II metabolism (Shaik, 2016). However, their application is to some extent limited because they are not readily available and difficult to reproduce the results due to high inter individual variability between human liver donors. In practice, human liver microsomes and hepatocytes are both applied for evaluating drug metabolism liability in order to get more detailed metabolism information for predicting *in vivo* human metabolite. Metabolite characterization continues to be a challenge for scientists. Ultra-high performance liquid chromatography coupled with Q (UHPLC-Q) Exactive Orbitrap mass spectrometer was demonstrated to be one of the most reliable techniques for metabolites characterization, which can provide accurate masses of metabolites and data dependent MS^2^ fragment ions for credible structural analysis (Lopez-Gutierrez et al., 2014; Scheidweiler and Huestis, 2014). The data processing software Metworks can easily find the potential metabolites according to mass defect filter (MDF) function and background subtraction program, which facilitates the identification of metabolites.

The current work aimed to identify the metabolite following incubation of evodiamine with human liver microsomes and hepatocytes by using UHPLC-Q Exactive mass spectrometer, and to propose the metabolic pathways of evodiamine in human. A total of 19 metabolites, including seven phase II metabolites, were detected and identified. Oxygenation, *N*-demethylation, GSH conjugation, and glucuronidation were the predominant metabolic pathways of evodiamine.

## Materials and Methods

### Chemicals and Reagents

Evodiamine with purity >98% was purchased from Shanghai PureOne BioTech Co., Ltd. (Shanghai, China). 10-Hydroxyevodiamine with purity >98% was purchased from Chengdu Herbpurify Co., Ltd. (Chengdu, China). 3-Hydroxyevodiamine, 3-hydroxyevodiamine glucuronide, and 10-hydroxyevodiamine glucuronide with the purity more than 97.5% were presented as a gift by Dr. Chunyong He and their structures were confirmed by high-resolution mass spectrometry and nuclear magnetic resonance spectroscopy. Cryopreserved human hepatocytes (20 donors) were purchased from the Research Institute for Liver Diseases (Shanghai) Co., Ltd. (Shanghai, China). Pooled human liver microsomes (20 donors) were purchased from BD Gentest (Woburn, MA, United States). NADPH and MgCl_2_⋅6H_2_O were purchased from Sigma–Aldrich (St. Louis, MO, United States). Deionized water was generated from Milli-Q Water Millipore Purification System (Millipore Corp., Bedford, MA, United States). All other chemicals and reagents were of analytical grade and commercially available.

### Metabolism of Evodiamine in Human Hepatocytes

Suspensions of human hepatocytes (1 × 10^6^ cell/ml) in Williams’ E medium were incubated for 2 h with 0.2% acetonitrile (control) or 10 μM evodiamine, rotating at 150 rpm at 37°C in an incubator at 5% CO_2_ and 95% humidity. The total volume of incubation was 200 μl. Viability of hepatocytes at pre-incubation and post-incubation were determined by trypan blue exclusion test and the viability was more than 80%. After incubation for 2 h, the biotransformation was terminated by adding 400 μl of acetonitrile, and then the samples were centrifuged at 12,000 rpm for 10 min. The resulting supernatant was evaporated to dryness under nitrogen gas at room temperature, and the residue was reconstituted with 200 μl of 20% acetonitrile. The sample was centrifuged at 12,000 rpm for 10 min. The resulting supernatant was transferred into a clear Eppendorf tube, and an aliquot of 5 μl was analyzed by LC/MS.

### Metabolism of Evodiamine in Pooled Human Liver Microsomes

All incubations were performed at 37°C in an incubator at 5% CO_2_ and 95% humidity. Stock solution of evodiamine was prepared in acetonitrile. The final concentration of acetonitrile in the incubation was 0.2% (*v/v*). The pooled human liver microsomes were carefully thawed on ice before experiment. Metabolism of evodiamine (10 μM) was performed using pooled human liver microsomes in 100 mM potassium phosphate buffer (pH 7.4), and the microsomal protein concentration was set at 0.5 mg/ml. After a 5-min pre-incubation at 37°C, the reactions were initiated by addition of cofactor solution containing 1.0 mM NADPH and 3.0 mM MgCl_2_. The total incubation volume was 400 μl. After incubation for 60 min, the reactions were terminated by adding 800 μl of acetonitrile, and then the samples were centrifuged at 12,000 rpm for 10 min. The resulting supernatant was evaporated to dryness under nitrogen gas at room temperature, and the residue was reconstituted with 200 μl of 20% acetonitrile. The sample was centrifuged at 12,000 rpm for 10 min. The resulting supernatant was then transferred into a clear Eppendorf tube, and a 5 μl portion was analyzed by LC/MS.

### UHPLC-Q Exactive Mass Spectrometer Conditions

The LC system consisted of a Thermo Dionex U3000 UHPLC system (Thermo Electron Corporation, San Jose, CA, United States), and chromatographic separations were carried out on an ACQUITY UPLC BEH C_18_ column (2.1 mm × 50 mm, i.d., 1.7 μm) thermostated at 40°C. The mobile phase consisted of 0.1% formic acid in water (A) and 0.1% formic acid in acetonitrile (B), at the flow rate of 0.3 ml/min. The gradient elution program was set as follow: 0–1 min, 10% B; 1–5 min, 10–40% B; 5–11 min, 40-55% B; 11–14 min, 55–90% B; 14–16 min, 90% B; and finally, the column was equilibrated with 10% B for 2 min. The sampler was kept at 10°C and the injection volume was 5 μl.

High-resolution MS and MS^2^ spectra were obtained on a Q-Exactive Orbitrap mass spectrometer (Thermo Electron Corporation, San Jose, CA, United States) equipped with an electrospray ionization interface operated in positive ion mode. The optimized parameters were set as follow: capillary voltage, 3.0 kV; sheath gas flow rate, 35 arbitrary unit; auxiliary gas flow rate, 5 arbitrary unit; sweep gas flow rate, 5 arbitrary unit; capillary temperature, 325°C; sheath gas heater temperature, 200°C. Data were acquired from 100 to 1000 Da with dd-MS^2^ or MS^2^ in centroid mode with ramp collision energy being set at 30, 35, and 45 eV. Raw data were acquired and processed using the Xcalibur software (Version 2.3.1, Thermo Electron Corporation, San Jose, CA, United States). Metworks software was used for post-acquisition data processing, which can automatically generate a list of proposed metabolites by comparing the LC/MS chromatograms of the drug-containing sample with the control samples according to MDF function.

## Results and Discussion

### Fragmentation of Evodiamine Standard

**FIGURE 2:**
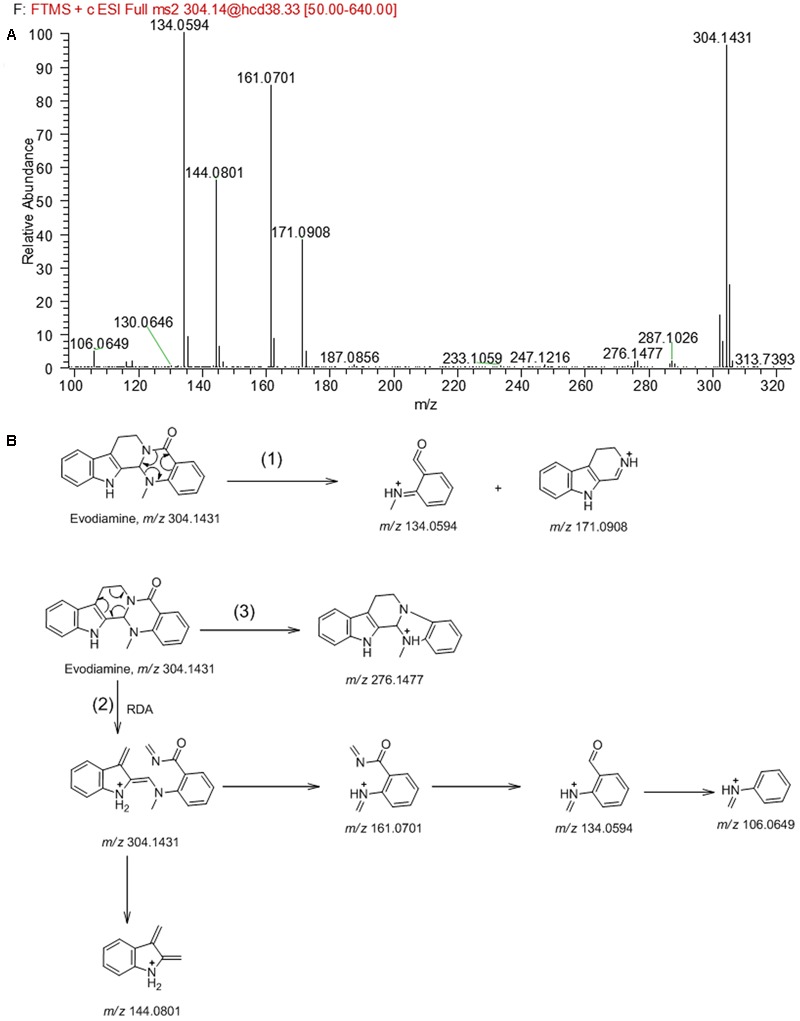
MS^2^ spectrum of evodiamine **(A)** and its fragmentation pathways **(B)** in positive ion mode.

In order to facilitate the structural identification of metabolites, the MS^2^ fragmentation behaviors of evodiamine was investigated by using UHPLC-Q Exactive mass spectrometer, which can provide accurate mass, element composition, and the error between calculated mass and measured mass. Evodiamine showed protonated ion [M+H]^+^ at *m/z* 304.1431 (-4.3 ppm, C_19_H_18_N_3_O) and produced a series of product ions in product ion scan. As shown in MS^2^ spectrum (**Figure 2A**), evodiamine showed characteristic product ions at *m/z* 276.1477, 171.0908, 161.0701, 144.0801, 134.0594, and 106.0649. The proposed fragmentation pathways of evodiamine were presented in **Figure 2B**. Among of them, the fragment ions at *m/z* 134.0594 and 171.0908 were attributed to the cleavage between the 2-aminobenzaldehyde and carboline moieties to form a benzoisoxazole after rearrangement. The fragment ions at *m/z* 161.0701 and 144.0801 were likely generated from ring fission through retro-Diels–Alder reaction followed by cleavage of C–N bond. The product ion at *m/z* 276.1477 was derived from the protonated ion *m/z* 304.1431 by loss of CO.

### LC/MS Analysis of Metabolites of Evodiamine

**FIGURE 3:**
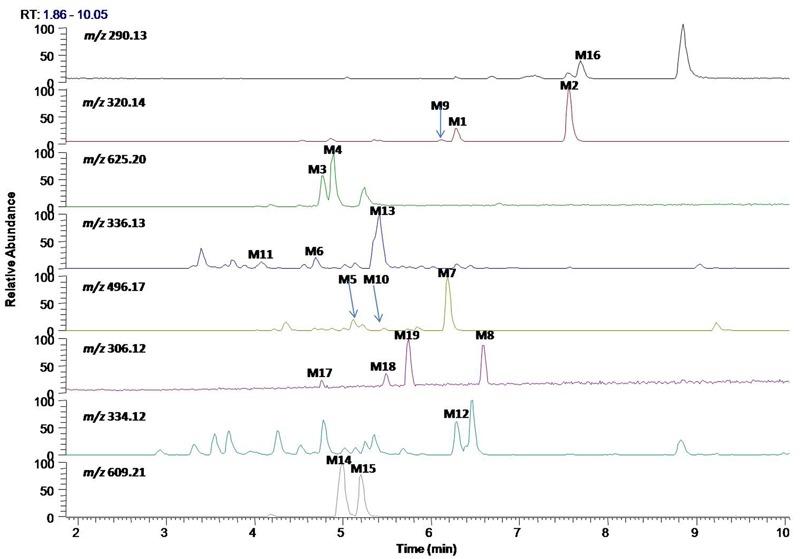
Extracted ion chromatograms of metabolites of evodiamine.

*In vitro* metabolites of evodiamine in human liver microsomes and hepatocytes were analyzed using LC/MS. The difference analysis between blank and evodiamine-containing incubation sample was performed by Metworks software (Thermo Electron Corporation, San Jose, CA, United States). Precursor ions specifically found in evodiamine-containing incubation samples were viewed as potential metabolites and were thus conducted for MS^2^ analysis. A total of 12 phase I metabolites were detected in human liver microsomes; whereas in human hepatocytes a total of 19 metabolites, including 7 phase II metabolites, were detected and identified. The extracted ion chromatograms of these metabolites are shown in **Figure 3**. The retention times, measured and theoretical masses, mass errors, and characteristic fragment ions of the proposed metabolites are summarized in **Table 1**. The maximum mass errors between the measured and theoretical values were within 5 ppm. The structures of metabolites were characterized based on their accurate masses, fragment ions, and retention times, and four metabolites (M1, M2, M5, and M7) were further confirmed by matching their retention times, accurate masses, and fragment ions with their reference standards.

**Table 1:** Characterization of *in vitro* metabolites of evodiamine by UHPLC-Q Exactive mass spectrometer.

Metabolites No.	RT (min)	Mass shift	Theo.	*m/z*	Meas. *m/z*	Error (ppm)	Fragment ions	Metabolic pathways
M1	6.27	15.9949	320.1394	320.1395	0.3	187.0851, 161.0693, 160.0745, 134.0591	Oxygenation (P + O)
M2	7.56	15.9949	320.1394	320.1395	0.3	177.0646, 171.0903, 150.0539, 144.0797, 122.0597	Oxygenation (P + O)
M3	4.78	321.0631	625.2075	625.2045	-4.8	550.1699, 496.1601, 492.1512, 363.1094, 336.0985, 219.0587, 192.0463, 134.0591	Oxygenation and GSH conjugation (P + O + C_10_H_15_N_3_O_6_S)
M4	4.90	321.0631	625.2075	625.2045	-4.8	550.1699, 496.1601, 492.1512, 363.1094, 336.0985, 219.0587, 192.0463, 134.0591	Oxygenation and GSH conjugation (P + O + C_10_H_15_N_3_O_6_S)
M5	5.12	192.0270	496.1714	496.1722	1.6	363.1160, 320.1394, 187.0850, 134.0591	Oxygenation and glucuronidation (P + O + C_6_H_8_O_6_)
M6	4.69	31.9898	336.1343	336.1343	0.0	318.1239,177.0645, 160.0745, 150.0538, 144.0797	Di-oxygenation (P + 2O)
M7	6.19	192.0270	496.1714	496.1722	1.6	344.0949, 326.0846, 320.1368, 171.0903, 150.0538, 144.0796	Oxygenation and glucuronidation (P + O + C_6_H_8_O_6_)
M8	6.61	1.9793	306.1237	306.1241	1.3	171.0905, 144.0799, 136.0384	*N*-demethylation and oxygenation (P - CH_2_ + O)
M9	6.09	15.9949	320.1394	320.1395	0.3	187.0851, 161.0693, 160.0745, 134.0591	Oxygenation (P + O)
M10	5.48	192.0270	496.1714	496.1722	1.6	363.1160, 320.1394, 187.0854, 134.0590	Oxygenation and glucuronidation (P + O + C_6_H_8_O_6_)
M11	4.07	31.9898	336.1343	336.1331	-3.6	318.1239, 176.0693, 161.0697, 134.0591	Di-oxygenation (P + 2O)
M12	6.29	29.9742	334.1186	334.1177	-2.7	174.0537, 161.0697, 134.0592	Di-oxygenation and dehydrogenation (P + 2O - 2H)
M13	5.41	31.9898	336.1343	336.1341	-0.6	187.0851, 176.0693, 161.0697, 134.0591	Di-oxygenation (P + 2O)
M14	5.00	305.0682	609.2126	609.2125	-0.2	480.1305, 308.0887, 302.1264, 179.0472	GSH conjugation (P + C_10_H_15_N_3_O_6_S)
M15	5.21	305.0682	609.2126	609.2125	-0.2	480.1305, 308.0887, 302.1264, 179.0472	GSH conjugation (P + C_10_H_15_N_3_O_6_S)
M16	7.69	-14.0157	290.1288	290.1281	-2.4	171.0905, 144.0799, 120.0436	*N*-demethylation (P - CH_2_)
M17	4.76	1.9793	306.1237	306.1241	1.3	187.0852, 160.0745, 120.0437	*N*-demethylation and oxygenation (P - CH_2_ + O)
M18	5.49	1.9793	306.1237	306.1241	1.3	187.0852, 160.0745, 120.0437	*N*-demethylation and oxygenation (P - CH_2_ + O)
M19	5.74	1.9793	306.1237	306.1241	1.3	187.0852, 160.0745, 120.0437	*N*-demethylation and oxygenation (P - CH_2_ + O)
Evodiamine	8.85	0.0000	304.1444	304.1431	-4.3	276.1477, 171.0908, 161.0701, 144.0801, 134.0594, 106.0649	Parent

*P, parent (evodiamine); Mass shift, mass different between metabolite and parent (evodiamine); Theo. m/z, theoretical mass charge ratio; Meas. m/z, measured mass charge ratio.*

### Metabolites M1 and M2

**FIGURE 4:**
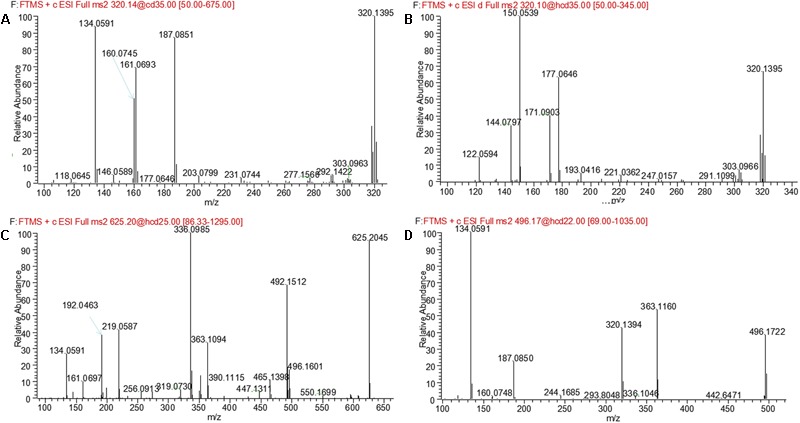
MS^2^ spectra of M1 **(A)**, M2 **(B)**, M3, and M4 **(C)**, and M5 **(D)**.

M1 and M2, eluted at the retention times of 6.27 and 7.56 min, respectively, showed an accurate protonated molecular ion at *m/z* 320.1395 (calculated 320.1394), 15.9964 Da higher than that of evodiamine, suggesting that M1 and M2 were oxygenation metabolites of evodiamine. MS^2^ spectrum of M1, as shown in **Figure 4A**, displayed a typical product ion at *m/z* 187.0851, suggesting that oxygenation occurred at carboline moiety. The other fragment ions at *m/z* 161.0693, 160.0745, and 134.0591 suggested that the 2-aminobenzaldehyde moiety remained unmodified. Compared with reference standard, the retention time, accurate mass, and product ions of M1 were identical to those of 10-hydroxyevodiamine. Hence, M1 was identified as 10-hydroxyevodiamine.

MS^2^ spectrum of M2 (**Figure 4B**) showed typical product ions at *m/z* 177.0676, 150.0539, and 122.0594, indicating that oxygenation occurred at the 2-aminobenzaldehyde moiety. The other fragment ions at *m/z* 171.0903 and 144.0797 were identical to those of parent, which suggested that carboline moiety remained intact. Compared with reference standard, the retention time, accurate mass and product ions of M2 were identical to those of 3-hydroxyevodiamine. Hence, M2 was identified as 3-hydroxyevodiamine.

### Metabolites M3 and M4

**FIGURE 5:**
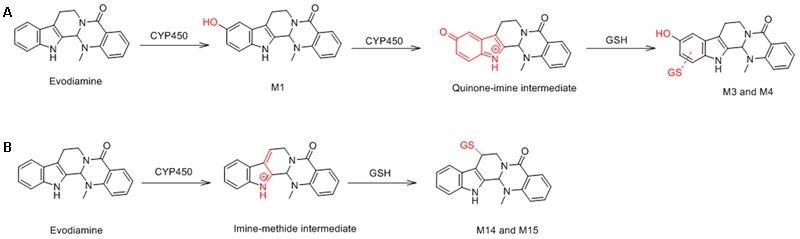
Formation of M3 and M4 **(A)** and M14 and M15 **(B)**.

M3 and M4 were eluted at 4.78 and 4.90 min, respectively. Both metabolites showed an accurate protonated molecular ion at *m/z* 625.2045 (calculated 625.2075), 321.0614 Da higher than that of evodiamine, suggesting that M3 and M4 were formed by oxygenation followed by GSH conjugation of evodiamine. MS^2^ spectra of M3 and M4 (**Figure 4C**) showed typical product ions at *m/z* 550.1699 and 496.1601, which were derived from neutral loss of glutamate residue (-129.0444 Da) and glycine residue (-75.0346 Da), respectively (Wen and Zhu, 2015). The product ions at *m/z* 492.1512, 363.1094, 336.0985, 219.0587, and 192.0463 demonstrated that oxygenation and GSH conjugation occurred at carboline moiety. It has been well-known that 5-hydroxyindole can form reactive species quinone-imine and this intermediate was readily conjugated with GSH (**Figure 5A**). Compared with previous report (Wen et al., 2014), M3 and M4 were therefore identified as GSH conjugates of M1.

### Metabolite M5

M5 was eluted at 5.12 min with accurate protonated molecular ion at *m/z* 496.1722 (calculated 496.1714), 192.0291 Da higher than that of parent, suggesting that M5 was formed by oxygenation followed by glucuronide conjugation of evodiamine. MS^2^ spectrum, as shown in **Figure 4D**, displayed a characteristic product ion at *m/z* 320.1394 which was formed via neutral loss of glucuronyl (-176.0328 Da). Product ions at *m/z* 363.1160, 187.0850, and 134.0591 suggested that oxygenation and glucuronidation occurred at carboline moiety. Compared with reference standard, the retention time, accurate mass, and product ions of M5 were identical to those of 10-hydroxyevodiamine glucuronide. M5 was hence identified as glucuronide conjugate of 10-hydroxyevodiamine (M1).

### Metabolite M6

**FIGURE 6:**
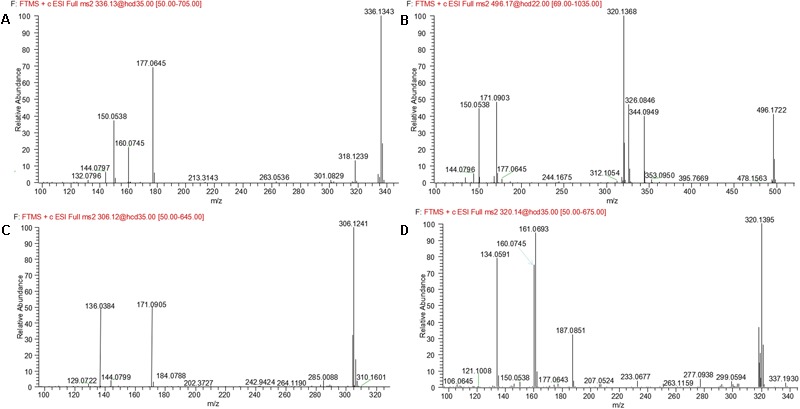
MS^2^ spectra of M6 **(A)**, M7 **(B)**, M8 **(C)**, and M9 **(D)**.

M6 was eluted at 4.69 min with an accurate protonated molecular ion at *m/z* 336.1343 (calculated 336.1343), 31.9912 Da higher than that of parent, suggesting that M6 was the di-oxygenation metabolite of evodiamine. MS^2^ spectrum of this precursor ion (**Figure 6A**) showed typical product ions at *m/z* 177.0645 and 150.0538, which suggested that one oxygenation occurred at 2-aminobenzaldehyde moiety. Considering that C-3 of evodiamine was readily hydroxylated, this oxygenation was likely to occur at C-3 position. Product ion at *m/z* 160.0745 suggested that other oxygenation occurred at carboline moiety. A minor product ion at *m/z* 318.1239 was formed by loss of H_2_O (-18.0104 Da) from precursor ion, which further demonstrated that oxygenation occurred at C-8 position of evodiamine. Therefore, M6 was derived from M2 via oxygenation.

### Metabolite M7

M7 was eluted at 6.19 min with an accurate protonated molecular ion at *m/z* 496.1722 (calculated 496.1714), 192.0291 Da higher than that of parent, suggesting that M7 was formed by oxygenation followed by glucuronide conjugation of evodiamine. MS^2^ spectrum of this precursor ion (**Figure 6B**) showed a characteristic product ion at *m/z* 320.1368, which was formed through neutral loss of glucuronyl (-176.0354 Da). Product ions at *m/z* 344.0949, 326.0846, 171.0903, and 150.0538 suggested that oxygenation and glucuronidation occurred at 2-aminobenzaldehyde moiety, and the carboline moiety remained unmodified. Compared with reference standard, the retention time, accurate mass and product ions of M7 were identical to those of 3-hydroxyevodiamine glucuronide. M7 was hence identified as glucuronide conjugate of 3-hydroxyevodiamine (M2).

### Metabolite M8

M8 was eluted at 6.61 min with an accurate protonated molecular ion at *m/z* 306.1241 (calculated 306.1237), 1.9810 Da higher than that of parent, suggesting that M8 was formed by oxygenation and demethylation of evodiamine. MS^2^ spectrum (**Figure 6C**) showed a characteristic product ion at *m/z* 171.0905 by loss of 2-aminobenzaldehyde moiety, suggesting that carboline moiety remained unmodified. Product ion at *m/z* 136.0384 proved that the oxygenation and demethylation occurred at 2-aminobenzaldehyde moiety. Considering that C-3 position of evodiamine was readily hydroxylated, M8 was tentatively formed from M2 via demethylation or from M16 via oxygenation.

### Metabolite M9

M9 eluted at the retention time of 6.09 min showed an accurate protonated molecular ion at *m/z* 320.1395 (calculated 320.1394), which was 15.9964 Da higher than that of parent, suggesting that M9 was oxygenation metabolite of evodiamine. MS^2^ spectrum of M9 (**Figure 6D**) showed typical product ion at *m/z* 187.0851, suggesting that oxygenation occurred at carboline moiety. The other product ions at *m/z* 161.0693, 160.0745, and 134.0591 suggested that the 2-aminobenzaldehyde moiety remained unmodified. Hence, M9 was identified as oxygenation metabolite of evodiamine.

### Metabolite M10

**FIGURE 7:**
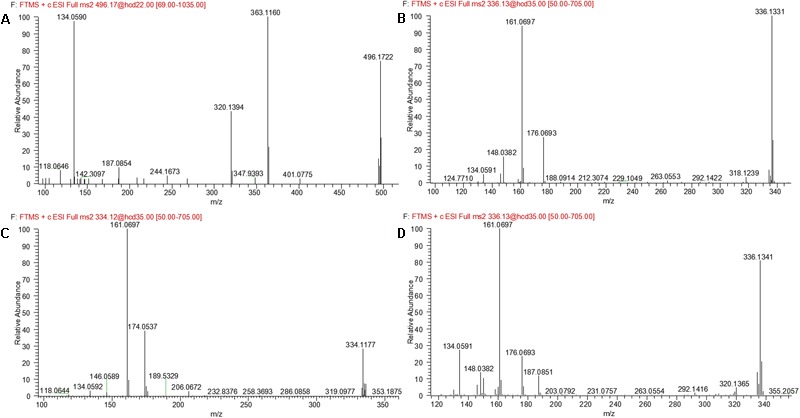
MS^2^ spectra of M10 **(A)**, M11 **(B)**, M12 **(C)**, and M13 **(D)**.

M10 was eluted at 5.48 min with an accurate protonated molecular ion at *m/z* 496.1722 (calculated 496.1714), 192.0291 Da higher than that of parent, suggesting that M10 was from oxygenation followed by glucuronide conjugation of parent. MS^2^ spectrum (**Figure 7A**) showed a characteristic neutral loss of glucuronyl (-176.0328 Da) to form product ion at *m/z* 320.1394. Product ions at *m/z* 363.1160, 187.0854, and 134.0590 suggested that oxygenation and glucuronidation occurred at carboline moiety. M10 was tentatively proposed as glucuronide conjugate of M9. This metabolic pathway was similar to that of rutaecarpine, an analog of evodiamine (Lee et al., 2017).

### Metabolite M11

M11 was eluted at 4.07 min with an accurate protonated molecular ion at *m/z* 336.1331 (calculated 336.1343), 31.9900 Da higher than that of parent, suggesting that M11 was derived from the di-oxygenation of parent. MS^2^ spectrum (**Figure 7B**) showed typical product ions at *m/z* 161.0697 and 134.0591, which suggested that 2-aminobenzaldehyde moiety remained intact. Product ion *m/z* 176.0693 indicated that di-oxygenation occurred at carboline moiety. A minor product ion at *m/z* 318.1239 was formed through loss of H_2_O (-18.0092 Da) from precursor ion, which further demonstrated that oxygenation occurred at C-8 position of evodiamine. Therefore, M11 was tentatively identified as the oxygenation metabolite of M1 or M9.

### Metabolite M12

M12 was eluted at 6.29 min with an accurate protonated molecular ion at *m/z* 334.1177 (calculated 334.1186), 29.9746 Da higher than that of parent, suggesting that M12 was derived from the di-oxygenation and dehydrogenation of parent. MS^2^ spectrum (**Figure 7C**) showed typical product ions at *m/z* 161.0697 and 134.0592, which suggested that 2-aminobenzaldehyde moiety remained unmodified. Product ion *m/z* 174.0537 indicated that di-oxygenation and dehydrogenation occurred at carboline moiety. Therefore, M12 was identified as dehydrogenation product of M11.

### Metabolite M13

M13 eluted at 5.41 min displayed an accurate protonated molecular ion at *m/z* 336.1341(calculated 336.1343), 31.9910 Da higher than that of parent, suggesting that M13 was from the di-oxygenation of parent. MS^2^ spectrum (**Figure 7D**) showed typical product ions at *m/z* 161.0697 and 134.0591, which suggested that 2-aminobenzaldehyde moiety remained unmodified. Product ion *m/z* 176.0693 indicated that di-oxygenation occurred at 3-methyleneindole moiety. A minor product ion at *m/z* 320.1365 was formed through loss of oxygen atom (-15.9976 Da) from precursor ion, which was a characteristic neutral loss of *N*-oxygenation metabolite. Therefore, M13 was tentatively identified as the *N*-oxygenation metabolite of M1 or M9.

### Metabolites M14 and M15

**FIGURE 8:**
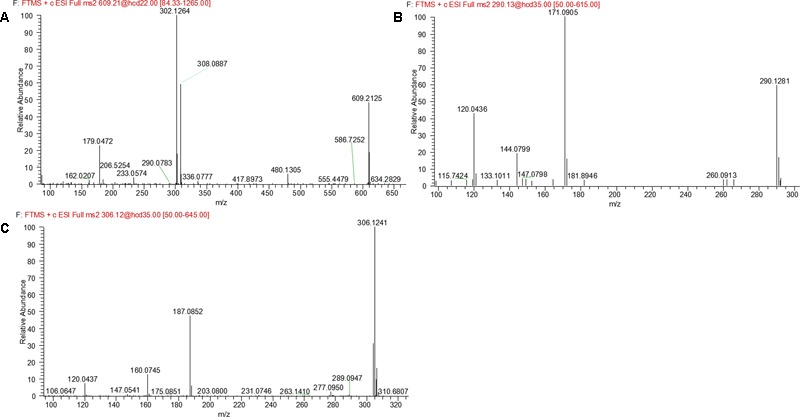
MS^2^ spectra of M14 and M15 **(A)**, M16 **(B)**, and M17–M19 **(C)**.

M14 and M15 were eluted at 5.00 and 5.21 min, respectively. Both metabolites showed an accurate protonated molecular ion at *m/z* 609.2125 (calculated 609.2126), 305.0694 Da higher than that of parent, suggesting that M14 and M15 were GSH conjugate of parent. MS^2^ spectra of M14 and M15 (**Figure 8A**) showed typical product ions at *m/z* 480.1305 and 302.1264, which were derived from neutral loss of glutamate residue (-129.0820 Da) and GSH residue (-307.0861 Da) (Wen and Zhu, 2015). Therefore, M14 and M15 were identified to be GSH conjugate of parent. Their formation may be through oxidation of 3-methyleneindole to form imine-methide intermediate, followed by conjugation with GSH as shown in **Figure 5B**.

### Metabolite M16

M16 was eluted at 7.69 min with an accurate protonated molecular ion at *m/z* 290.1281 (calculated 290.1288), 14.0150 Da lower than that of parent, suggesting that M16 was the *N*-demethylation metabolite of evodiamine. MS^2^ spectrum (**Figure 8B**) showed typical product ions at *m/z* 171.0905 and 144.0799, which were identical to those of parent. Product ion at *m/z* 120.0436 further proved that M16 was the *N*-demethylation metabolite of parent (Sun et al., 2013).

### Metabolite M17–M19

M17, M18, and M19 were eluted at 4.76, 5.49, and 5.74 min, respectively. They all showed an exact protonated molecular ion at *m/z* 306.1241 (calculated 306.1237), 15.9960 Da higher than that of M16, suggesting that these metabolites were derived from oxygenation of M16. MS^2^ spectrum (**Figure 8C**) showed characteristic product ions at *m/z* 187.0852 and 160.0745, suggesting that oxygenation occurred at 3-methyleneindole moiety. Therefore, M17, M18, and M19 were identified as oxygenation products of M16.

### *In Vitro* Metabolism of Evodiamine

**FIGURE 9:**
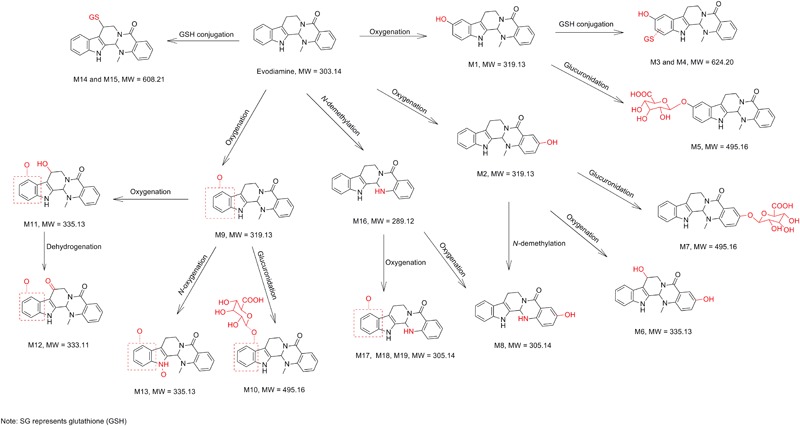
Proposed metabolic pathways of evodiamine in human hepatocytes and liver microsomes.

*In vitro* metabolism of evodiamine in human liver microsomes and hepatocytes were investigated using UHPLC-Q Exactive mass spectrometer and the metabolic pathways were accordingly proposed, as shown in **Figure 9**. In general, the *in vitro* metabolism of evodiamine can be concluded to undergo four pathways. The first pathway is oxidation of indole moiety to form oxygenated metabolites M1 and M9, which undergo further metabolism to form GSH conjugates (M3 and M4) via reactive quinone-imine intermediate, to form glucuronide conjugates (M5 and M10), and to form di-oxygenation metabolites (M11 and M13). The second pathway is oxidation of C-3 position to form M2, which were further metabolized via oxygenation, glucuronidation and *N*-demethylation to form M6, M7, and M8, respectively. The third metabolic pathway is *N*-demethylation to form M16, which was further metabolized into oxygenated metabolites (M8, M17, M18, and M19). The fourth metabolic pathway is direct conjugation with GSH via reactive imine-methide intermediate to form GSH adducts (M14 and M15). Therefore, oxygenation, demethylation, GSH conjugation, and glucuronidation were the predominant metabolic pathways of evodiamine in human liver microsomes and hepatocytes.

There are several literatures regarding metabolism of alkaloids from *E. rutaecarpa* (Li et al., 2006; Wen et al., 2014; Lee et al., 2017). Due the lack of references, the major metabolic sites were not assigned. Previous report by Wen et al. (2014) mainly focused on metabolic bioactivation of evodiamine. The author concluded that the formation of reactive metabolites is associated with the moderate hepatotoxicity of evodiamine. However, it lacks the overall metabolite profiles of evodiamine and the conclusion might be a little arbitrary because reactive metabolites do not always mean toxicity, such as cyclobenzaprine (Kalgutkar and Dalvie, 2015). The relationship between reactive metabolites and hepatotoxicity is very complex and hard to be established. A lot of factors can affect the hepatotoxicity, such as dose, multiple metabolic pathways, genetic polymorphism, and the amount of reactive metabolite *in vivo*. Sun et al. (2013), identified several hydroxylated metabolites of evodiamine *in vitro*, but they missed the phase II metabolites. Our study provided detailed metabolite profiles of evodiamine in human liver microsomes and hepatocytes, leading to 12 new metabolites being identified (M2, M5–M8, M10–M13, and M17–M19). Our findings suggested that evodiamine undergo multiple metabolic pathways, which is helpful in understanding the mechanism of toxification and detoxification of evodiamine. Furthermore, our study provides valuable information in predicting *in vivo* human metabolite. Future study will be intended to focus on the covalent binding of evodiamine to protein and safety assessments of the newly identified metabolites.

## Conclusion

In the present study, a rapid and reliable analytical method based on UHPLC-Q-Orbitrap-MS was developed and used for the identification of evodiamine metabolites in human liver microsomes and hepatocytes. A total of 12 phase I metabolites were detected in human liver microsomes; whereas in human hepatocytes a total of 19 metabolites, including seven phase II metabolites were detected. Four metabolites (M1, M2, M5, and M7) were further unambiguously confirmed by matching their retention times, accurate masses and fragment ions with their reference standards. Evodiamine was proved to be metabolized mainly via oxygenation, *N*-demethylation, glucuronidation, and GSH conjugation. Findings from the current work are helpful in understanding the mechanism of toxification and detoxification of evodiamine. Furthermore, our study provides valuable information in predicting *in vivo* human metabolites.

## Author Contributions

ZZ, JY, and QL conceived and designed the experiments. ZZ, TF, and HZ performed the experiments. ZZ, JY, and QL analyzed the data. ZZ and JY contributed reagents/materials/analysis tools. ZZ, JY, and QL wrote the paper.

## Conflict of Interest Statement

The authors declare that the research was conducted in the absence of any commercial or financial relationships that could be construed as a potential conflict of interest.
